# Insomnia symptom severity and dynamics of arousal‐related symptoms across the day

**DOI:** 10.1111/jsr.14276

**Published:** 2024-06-25

**Authors:** Leonie J. T. Balter, Eus J. W. van Someren, John Axelsson

**Affiliations:** ^1^ Department of Clinical Neuroscience Karolinska Institutet Stockholm Sweden; ^2^ Department of Psychology Stress Research Institute, Stockholm University Stockholm Sweden; ^3^ Department of Sleep and Cognition Netherlands Institute for Neuroscience Amsterdam The Netherlands; ^4^ Department of Integrative Neurophysiology and Psychiatry, Center for Neurogenomics and Cognitive Research, Amsterdam UMC, Amsterdam Neuroscience VU University Amsterdam The Netherlands

**Keywords:** arousal, diurnal patterns, dynamics, insomnia symptoms

## Abstract

Arousal is a central component of many emotional symptoms and can contribute to insomnia. Here we assessed how the timing and fluctuating nature of arousal‐related symptoms over the course of the day relate to insomnia symptom severity. In this study, 361 participants (*M* age = 31.9 years, 282 women, 77 men, 2 non‐binary individuals) completed the Insomnia Severity Index to assess severity of insomnia symptoms, followed by repeated ratings of anxiety or nervousness, stress, sleepiness, and feeling down via their mobile phone between ~08:00 hours and 00:00 hours across 1 day. Measures of dynamics included: mean levels across the day; variation (standard deviation); instability (mean squared successive differences); and resistance to change/inertia (first‐order autocorrelation). Time‐of‐day patterns were modelled using generalized additive mixed effects models. Insomnia symptom severity (mean Insomnia Severity Index = 9.1, SD = 5.2, range 0–25) was associated with higher mean levels of all arousal‐related symptoms, and increased instability and variation throughout the day in anxiety or nervousness, stress, and feeling down. Resistance to change (inertia) was not associated with insomnia symptom severity. Generalized additive mixed effects analyses showed that while individuals with more severe insomnia symptoms had elevated symptoms across the entire day, they were especially more anxious or nervous and sleepy in the early morning (~08:00 hours), anxious or nervous, stressed and sleepy in the late afternoon/early evening (~16:00 hours–21:00 hours), and anxious or nervous and stressed in the late evening (~22:00 hours). Remarkably, higher arousal occurred in the presence of high subjective sleepiness. Together these results indicate that insomnia symptom severity is associated with problems with daytime and evening arousal regulation.

## 1 INTRODUCTION

Symptoms of insomnia such as problems with sleep initiation, maintenance and non‐restorative sleep are widely experienced among the general population (Biddle et al., 2018; Morin et al., 2006; Roth, 2007). In addition to night‐time symptoms, insomnia is associated with daytime impairments and emotional distress, in particular elevated levels of depression, anxiety and stress symptoms (Jansson‐Fröjmark & Lindblom, 2008; Riemann et al., 2022; Roth, 2007).

Problematic emotional patterns, encompassing challenges with the frequency, intensity, expression, regulation and/or timing of emotions, are common in various forms of mental illness (Gross & Jazaieri, 2014). The dynamics of emotions—the pattern of variation that unfolds from moment to moment—have been found to play a role in the onset and persistence of bipolar and depressive psychopathology (Kuppens et al., 2012; Sperry et al., 2020). Heightened negative affect reactivity, even when minor, is thought to have a cumulative impact on mental well‐being (Wichers et al., 2015). Such heightened reactivity may contribute to maladaptive cognitive and behavioural patterns that can further exacerbate insomnia symptoms (Kalmbach et al., 2018). Within the context of emotion dynamics, mean levels provide a measure of the average state or intensity over a given period. Emotional variability refers to the general spread of an emotion across the day and is often measured using the standard deviation (SD). Instability refers to the degree to which emotions change or fluctuate from one moment to the next, capturing variations and rapid changes, and is commonly assessed using the mean squared successive difference (MSSD) (Jahng et al., 2008). In addition to variability and instability, emotions can also be resistant to change. This resistance, or “emotional inertia”, is measured by autocorrelation, which indicates the likelihood of staying in a specific state over time, suggesting a failure to downregulate and return to baseline emotion levels (Koval et al., 2015). A meta‐analysis on emotion dynamics shows that subclinical and clinical levels of affect disorders are associated with more variable, unstable and inert negative emotions (Houben et al., 2015). While insomnia has been linked to emotion regulation difficulties, increased negative affect and elevated risk for affect disorders (Hertenstein et al., 2019; Jansson‐Fröjmark et al., 2016; Meneo et al., 2023; Van Someren, 2021), little attention has been given to its relation to dynamics of mental states.

Particularly relevant for understanding insomnia are the dynamics of arousal‐related symptoms such as anxiety and stress (associated with elevated physiological arousal), and sadness and sleepiness (associated with low physiological arousal) (Kreibig, 2010). The hyperarousal theory of insomnia suggests that a persistent state of physiological and cognitive arousal contributes to insomnia (Riemann et al., 2010). Aligning with this theory, symptoms consistent with heightened daytime and night‐time arousal, such as rumination and worry, are commonly observed in insomnia (Kalmbach et al., 2020; Meneo et al., 2023). These symptoms can interfere with the ability to fall asleep, as observed in studies involving university students with elevated depression symptoms (Pillai et al., 2014) and individuals who ruminated more after exposure to a psychosocial stressor (Zoccola et al., 2009). Moreover, a network analysis identified trouble relaxing as a central symptom connecting insomnia, depression and anxiety (Bard et al., 2023), suggesting arousal dysregulation as a potential research target. Besides elevated basal levels of arousal symptoms, insomnia has been related to stronger temporal variability in neural activity (Meng et al., 2020), which may contribute to stronger variability in the capacity to maintain alertness during wake states or maintain uninterrupted sleep throughout the night. Stronger day‐to‐day variability in mood, energy, concentration and alertness has been reported in a pilot study of seven individuals with insomnia versus eight controls (Levitt et al., 2004). Another study including 47 individuals with insomnia and 18 controls found stronger day‐to‐day variability in sleepiness but not alertness, positive mood or negative mood (Buysse et al., 2007). Together these data suggest that individuals with insomnia may experience increased day‐to‐day variability in their symptoms and daytime functioning. There is, however, a lack of studies assessing arousal‐related symptoms and fluctuations within a shorter timeframe, notably across the day. Moreover, there is limited research into how arousal‐related symptoms develop across the day, specifically with regard to individual differences in severity of insomnia symptoms. In the current study, we assessed the dynamics of arousal‐related symptoms across the day between ~08:00 hours and 00:00 hours in a population with varying degrees of insomnia symptom severity. Specifically, we assessed the dynamics and diurnal patterns of anxiety or nervousness, stress, sleepiness, and feeling down.

## 2 MATERIALS AND METHODS

### 2.1 Participants

The final analysis included 361 participants (*M* age = 31.9 years, SD = 8.9, age range 18–63 years, *n* = 282 women, 77 men, 2 non‐binary individuals). Twenty‐eight participants were excluded due to completing fewer than three out of six assessments, of which six failed to pass the data quality check. This check involved two attention questions and one honesty question: “Please rate the response alternative ‘agree (9)’ for this question”; “Please answer 100”; and “Have you been completely honest in your answers?”. Data of participants who failed more than one quality check were excluded. Participants were recruited via an online recruitment platform for academic research (Prolific.co), were fluent in English, resided in the UK, and were 18 years old or older. The study was advertised on the Prolific website, and only participants with a Prolific account could participate. No other inclusion or exclusion criteria applied, in order to recruit participants with a diverse range of psychiatric and insomnia symptom levels. Studies that have evaluated data quality of online recruitment platforms indicate that the Prolific platform delivers high‐quality data in terms of attention, comprehension, honesty and reliability (Douglas et al., 2023; Peer et al., 2022). Compensation was £7.50 per hour with a bonus of £5 upon completion of all sessions.

### 2.2 Study design

The study consisted of seven sessions across 2 days (weekdays only). On day 1, a baseline assessment was completed between ~09:00 hours and 21:00 hours during which the Insomnia Severity Index (ISI) was administered. On day 2, arousal symptoms were rated six times between ~08:00 hours and 23:00 hours. All were instructed to start the first assessment of day 2 any time between 08:00 hours and 09:00 hours. This start time was self‐chosen to minimize the study's interference with the participant's habitual sleep schedule prior to the ratings. Participants were encouraged to start at the instructed times for subsequent assessments, but each respective assessment could be completed until after the instructed times. This means that data points are recorded until after the start time of the last assessment (23:00 hours). The study included additional measures, such as a set of psychiatric disorder questionnaires administered at baseline (day 1) and psychiatric symptoms ratings across the day (day 2). The results from these measures are not reported here (see Balter et al., 2024). Data collection was conducted via the participants' personal smartphones. All data were time stamped and the time stamp was used for the analysis. See the Supplement for additional details about the study procedures. The study was approved by the Swedish Ethical Review Authority (dnr: 2021–01695). Participants provided online informed consent at the start of the baseline session.

### 2.3 Measures

#### 2.3.1 Insomnia

The ISI is a seven‐item scale that has high diagnostic accuracy (Bastien et al., 2001). It evaluates the nature, severity and impact of insomnia problems experienced in the past 2 weeks. Items are rated on a five‐point scale from “0 = no problem” to “4 = very severe”, yielding a total continuous score ranging between 0 and 28. An ISI score of 0‐7 is suggestive of no clinically significant insomnia, 8‐14 of subthreshold insomnia, 15‐21 of clinical insomnia with moderate severity, and 22‐28 of clinical insomnia with severe severity.

### 2.4 Arousal‐related symptoms

The current analysis focused on four items. The items “Have you felt nervous or anxious?”, “Have you felt stressed?”, and “Have you felt down?” were rated regarding “this morning” (Day 2, assessment 1), “since the last session” (Day 2, assessment 2), or in the past 3 hr (all other assessments on Day 2). Sleepiness was assessed with the question “How sleepy are you?”. All items were rated from “1 = Not at all” to “9 = Very much/All the time”, and rescored to 0 to 8 for the analyses.

## 3 STATISTICAL ANALYSIS

### 3.1 Dynamics of arousal‐related symptoms

For each arousal symptom, four outcomes were calculated: mean level (mean); variability (SD); instability (MSSD); and resistance to change (autocorrelation function [ACF]). First, mean levels were calculated to provide a measure of the average state or intensity across the day, calculated as an average score across all day 2 assessments. Second, within‐person variability across the day was assessed using the SD. The higher the SD, the stronger the variation. Third, the MSSD was calculated as a measure of instability, estimated using the *mssd* function of the *REAT* R package (Wieland, 2019). The MSSD calculates the average absolute differences between temporally adjacent ratings (squared differences between consecutive data points, for all pairs of consecutive data points). A higher MSSD value suggests that a person's state is more prone to rapid and pronounced fluctuations over time. Fourth, to capture the degree of resistance to change, the first‐order autocorrelation was calculated, indicating the likelihood of staying in a specific state from one observation (t0) to the next (t1), also referred to as “inertia” (Trull et al., 2015). To that extent, the *acf* function of the *stats* base R package was used. This function calculates a correlation coefficient that shows how closely the values in a series at one time point are related to the values at the previous time point (lag *t* − 1). The computation involves normalizing the values by dividing them by the variance of the series. The resulting inertia score can range from −1 to 1. A higher positive score indicates a tendency for the values to follow their past values, meaning if a value is higher than average at time *t* − 1, it is more likely to be followed by another higher value, and vice versa for lower values. Conversely, a negative score suggests that the variable tends to oscillate around its average value. If the score at *t* − 1 is above the average, it becomes more likely for the score at time *t* to be below average, and vice versa.

These measures of dynamics, i.e. SD, MSSD and ACF, are mathematically related to each other (Jahng et al., 2008), which highlights the relevance of studying them in conjunction (Dejonckheere et al., 2019; see Figure S2 for a correlation matrix of the outcome measures).

To assess whether these dynamics measures differ as a function of ISI (treated as a continuous variable), fixed effect regression analyses were performed, using the *lm* function in R. Outlier values, defined as values exceeding 4*SD* above or below the sample means, were removed. Fifteen out of 5776 (0.26%) datapoints exceeded this (3 for MSSD anxiety or nervousness; 4 for MSSD stress; 2 for MSSD sleepiness; 6 for MSSD down).

### 3.2 Modelling time‐of‐day patterns of arousal symptoms

To examine how arousal symptoms change throughout the day and whether these patterns vary based on the individual's insomnia symptom level, generalized additive mixed effects models (GAMMs) were used. GAMM is well‐suited for capturing non‐linear relationships, facilitating an understanding of the complexities of how arousal‐related symptoms develop across the day. Two models were formulated. The first model included a random intercept for participant ID using a ridge penalty and smooth terms for ISI and time‐of‐day (continuous time stamped data) to examine how the item is associated with insomnia symptom severity and how the item varies over the day, respectively. The second model additionally added a tensor product interaction smooth term of ISI by time‐of‐day to examine how the effect of time‐of‐day on the item varies depending on ISI. The best‐fitting response distributions were assessed based on the Akaike information criterion (AIC) score comparisons. Model comparison results can be found in Figure S4. The *bam* function of the *mgcv* R package (Wood, 2017) with the Poisson distribution with a log link function or a Gaussian distribution was used. To estimate the smoothing parameters, a penalized likelihood method with a fast restricted maximum likelihood was used. To reduce the risk of overfitting, double penalty shrinkage was used on the predictors. This means that if a given fixed‐effect predictor did not meaningfully contribute to the model, its effect was shrunk toward zero. The temporal patterns are interpreted by visualizing model predictions. Missing data (703 [8.8%] missing values out of 7961 arousal ratings) were imputed using the *k*‐nearest neighbours (kNN) function of the VIM R package (Kowarik & Templ, 2016). To incorporate the temporal patterns in the data, observations with a similar time‐of‐day were given higher weights in the imputation process. The four nearest neighbours were used for imputing missing values. See Figure S1 for density plots of observed and imputed data for each variable.

## 4 RESULTS

### 4.1 Dynamics of arousal‐related symptoms

**FIGURE 1:**
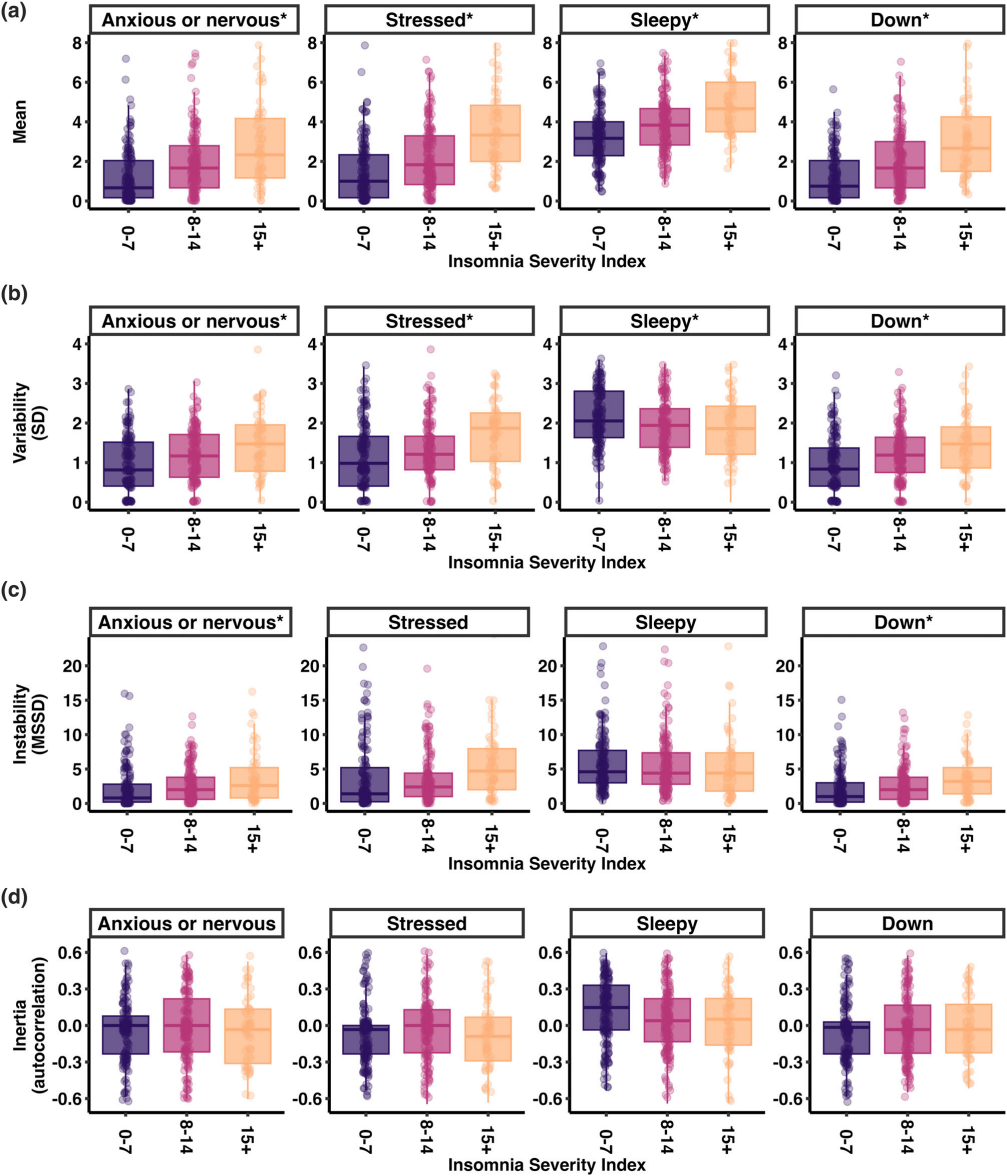
Boxplots illustrating the relationship between Insomnia Severity Index (ISI) scores and: (a) mean levels across the day; (b) variability measured using the standard deviation (SD); (c) instability measured using the mean squared successive differences (MSSD); and (d) inertia measured by autocorrelation. For visualization purposes, each boxplot represents the mean value for individuals with ISI scores in the following ranges: 0–7 ISI score (no clinically significant insomnia, *N* = 152); 8–14 (subthreshold insomnia levels, *N* = 154); 15 or higher (moderate to severe clinical levels of insomnia, *N* = 55). An asterisk in the title indicates a statistically significant relationship between ISI and the respective outcome. Individual datapoints are denoted by jittered dots in each boxplot.

Sample distribution plots of ISI scores (*M* = 9.1, SD = 5.2, range 0–25) can be found in Figure S3. As shown in Figure 1 and Table 1, higher ISI scores were associated with higher mean levels of anxiety or nervousness, stress, sleepiness, and feeling down, as well as stronger variability (SD) and instability (MSSD) in anxiety or nervousness, stress, and feeling down, except for sleepiness (less variability and no significant relationship with instability). Inertia (resistance to change) was not associated with ISI for any of the items.

**TABLE 1:** Linear regression results of the relationships between ISI and dynamics of arousal‐related symptoms including mean levels, variability (SD), instability (MSSD) and inertia (ACF)

Variable	*b*	SE	95% CI		
			Lower bound	Upper bound	*p*
Mean Anxious or nervous***	0.118	0.016	0.087	0.148	0.000
Mean Stressed***	0.140	0.016	0.108	0.172	0.000
Mean Sleepy***	0.115	0.014	0.088	0.142	0.000
Mean Down***	0.131	0.015	0.102	0.160	0.000
SD Anxious or nervous***	0.038	0.007	0.024	0.052	0.000
SD Stressed***	0.037	0.008	0.020	0.053	0.000
SD Sleepy**	−0.020	0.007	−0.035	−0.006	0.005
SD Down***	0.038	0.007	0.024	0.053	0.000
MSSD Anxious or nervous***	0.106	0.030	0.046	0.165	0.001
MSSD Stressed	0.088	0.045	0.000	0.175	0.051
MSSD Sleepy	−0.036	0.043	−0.120	0.048	0.399
MSSD Down**	0.090	0.029	0.035	0.146	0.002
ACF Anxious or nervous	0.000	0.003	−0.005	0.006	0.949
ACF Stressed	0.002	0.003	−0.003	0.007	0.474
ACF Sleepy	−0.004	0.003	−0.009	0.002	0.188
ACF Down	0.003	0.003	−0.002	0.008	0.252

*Note*: The change in *b* represents the change in the outcome when ISI increases with one scale step.

Abbreviations: ACF, autocorrelation function; *b*, unstandardized coefficient; CI, confidence interval; MSSD, mean squared successive differences; SD, standard deviation; SE, standard error.

*** *p* < 0.001.

** *p* < 0.010.

### 4.2 Time‐of‐day patterns of arousal symptoms

**FIGURE 2:**
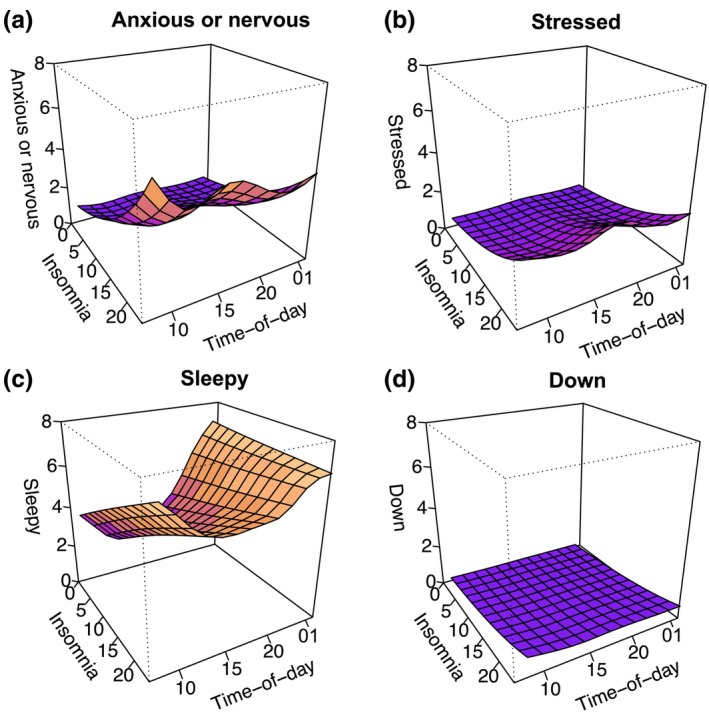
Three‐dimensional plot of predicted values of: (a) anxious or nervous; (b) stressed; (c) sleepy; and (d) down as a function of time‐of‐day and insomnia (Insomnia Severity Index [ISI]). The colour gradient represents the predicted intensity of the item, with warmer colours indicating a higher intensity on the *y*‐scale. The plots are based on a grid of 12 × 12 values, where each box represents a 2‐point increase in insomnia severity score and a ~2‐hr difference in time‐of‐day (time‐of‐day starts at 08:00 hours). All ratings were made on a 1–9 scale but here rescaled to 0–8, where 0 = not at all and 8 = very much/all the time.

The three‐dimensional GAMM predictions are visualized in Figure 2. All items non‐linearly changed across the day, as indicated by main effects of time‐of‐day. The highest level of anxiety or nervousness was observed in the early morning (~08:00 hours) and evening (~19:00 hours and later). The peak stress level occurred in the evening (~19:00 hours). Sleepiness was most pronounced in the early morning (~08:00 hours) and particularly in the late evening. Feeling down showed negligible variation across the day on the group level. While those with higher ISI scores reported higher symptom levels across the entire day than those with lower ISI scores, they experienced particularly higher levels of anxiety or nervousness in the early morning (~08:00 hours) and late afternoon/evening (~16:00‐19:00 hours and later), higher stress levels in the early morning (~08:00 hours) and early and late evening (~19:00 hours and ~01:00 hours), as well as higher sleepiness levels in the morning and afternoon (~08:00 hours–18:00 hours). Sleepiness levels were similar across ISI scores in the late evening, while anxiety or nervousness and stress levels were still elevated as compared with those with lower ISI scores. Except for sleepiness, individuals with low ISI scores experienced minimal other arousal symptoms throughout the day. The GAMM results are reported in Tables S1–S4 (anxious or nervous, stressed, sleepy, and down, respectively).

## 5 DISCUSSION

In the present study we characterized how arousal‐related symptoms vary over the course of the day in individuals with different levels of insomnia symptoms. Participants with more severe insomnia symptoms experienced higher mean levels of anxiety or nervousness, stress, sleepiness, and feeling down across the day. They also showed stronger variability in anxiety or nervousness, stress, and feeling down across the day, but lower variability in sleepiness. More severe insomnia symptoms were also associated with less stability in anxiety or nervousness, stress, and feeling down. Individuals with more severe insomnia symptoms were especially more anxious or nervous and sleepy in the early morning, and anxious or nervous, stressed, and sleepy in the late afternoon/evening. Insomnia symptom severity was not related to resistance to change (inertia) in arousal‐related symptoms. These results show that more severe insomnia symptoms are not only related to higher levels of arousal‐related experiences throughout the day but also more pronounced fluctuations.

In support of the hyperarousal theory of insomnia, our findings reveal that the severity of insomnia symptoms is associated with more pronounced symptoms of arousal throughout the day. This includes symptoms of hyperarousal such as anxiety and stress as well as symptoms associated with hypoarousal, i.e. sleepiness and feeling down. Sleep experts distinguish sleepiness, which relates to the ability to fall asleep, from tiredness/fatigue, encompassing a broader sense of weariness or lack of energy including both physical and mental aspects (Raizen et al., 2023). However, in everyday language, people often perceive them as similar, unless specific differentiating questions are used (Singareddy et al., 2010). The phenomenon of feeling simultaneously sleepy/tired and aroused in our sample may be referred to as “tired but wired” (Miglis, 2016), suggesting that high and low arousal symptoms can appear in parallel. While this might seem contradictory, there are also data showing that during stressful periods, individuals typically experience more sleepiness (Dahlgren et al., 2005). This could be related to the presence of higher homeostatic sleep pressure, resulting from insufficient sleep or a stress‐induced increase in sleep need. These factors could contribute to an overaction of the sympathetic branch of the autonomic nervous system (ANS), contributing to feelings of sleepiness (Donadio et al., 2007), which serve the purpose of reducing metabolism and motivating the individual to ensure recovery sleep (Axelsson et al., 2020). To remain awake and function under the extra homeostatic sleep pressure and sleepiness, the ANS would need to stimulate the parasympathetic branch of the ANS, resulting in hyperarousal states. This state of co‐activation of the parasympathetic and sympathetic branch may manifest as simultaneously experiencing high and low arousal symptoms and stronger variability in symptoms. Future research may elucidate the role of the ANS in the fluctuations of arousal symptoms and their contribution to insomnia.

The results further identified distinct patterns of arousal‐related symptoms, where individuals with elevated insomnia symptoms experienced peaks in arousal‐related symptoms during specific times of the day, including early morning (sleepiness and anxiety) and late afternoon/evening (anxiety and stress). The understanding of temporal patterns of symptoms holds promise for precision medicine and implementation of tailored chronotherapeutic approaches to address specific symptom fluctuations, where interventions could be strategically timed. An intriguing research question arising from the current study is whether targeting the morning peak in anxiety symptoms could induce a cascading effect, aimed at alleviating the anxiety and stress peaks in the late afternoon and evening, ultimately contributing to improved sleep. Such a focused intervention aligns with the concept of chrono‐treatments, which seeks to leverage the body's rhythms to enhance therapeutic outcomes (Lee et al., 2021). The presented findings offer a compelling foundation to further investigate time‐of‐day variation in arousal‐related symptoms and its implication for insomnia management.

Our finding of stronger instability in affect and arousal symptoms with insomnia symptoms parallels research demonstrating the significance of emotional instability in the onset of bipolar and depression disorder (Eldesouky et al., 2018; Sperry et al., 2020). Considering that both insomnia and depression are associated with persistent hyperactivity in the arousal system (Riemann et al., 2020), delving deeper into the intertwined nature of insomnia and depression with affect and arousal instability may shed light on shared underlying mechanisms. Further longitudinal investigations may explore the temporal development of insomnia and depression disorder, tracking how patterns of affect and arousal dynamics evolve over time.

A limitation of this study is the limited control of the participants' surroundings when completing the ratings. Future research may also assess the stability of the effects, for example by assessing time‐of‐day variation over multiple days, as well as how findings translate to clinical populations of insomnia. Lastly, the study assessed fluctuations in arousal symptoms within the day over multiple hours. It has been suggested that stronger variability over longer timescales, i.e. several hours or days, are a state of volatility, while fluctuations on a shorter timescale (i.e. seconds or minutes) may reflect functional responsiveness to situational contingencies (Koval et al., 2016). Future research may address the tipping point at which the changes in arousal symptoms transition from being adaptive to being maladaptive, and to what degree they relate to the aetiology of insomnia.

To conclude, the current results suggest problems with daytime and evening arousal regulation in insomnia, where insomnia symptom severity was generally related to higher arousal levels, particularly in the morning hours and early evening, as well as stronger fluctuations of both low and high arousal‐related symptoms. Notably, the increased arousal occurs in the presence of simultaneously increased sleepiness.

## AUTHOR CONTRIBUTIONS

**Leonie J. T. Balter:** Conceptualization; investigation; funding acquisition; writing – original draft; methodology; visualization; writing – review and editing; formal analysis; project administration; data curation. **Eus J. W. van Someren:** Conceptualization; writing – review and editing. **John Axelsson:** Conceptualization; writing – review and editing; funding acquisition.

## FUNDING INFORMATION

This work is supported by Rut and Arvid Wolff Memorial Foundation (LJTB) and SU‐Region Stockholm (FoUI‐980356; LJTB and JA). EJWVS work is supported by funding from the European Research Council (ERC), Brussels, Belgium, Advanced Grant ERC‐ADG‐2021‐101055383‐OVERNIGHT.

## CONFLICT OF INTEREST STATEMENT

The other authors declare that they have no competing interests.

## Supporting information


**DATA S1.** Supporting Information.

## Data Availability

The data are available upon request from the corresponding author. The analysis script notebook can be found on the Open Science Framework via https://osf.io/fmhtc/files/osfstorage/66732cdcb5a03601629ff60d.
