# Supplementary material for: Insomnia symptom severity and dynamics of arousal‐related symptoms across the day
Source: J Sleep Res. 2024 Jun 25;34(1):e14276. doi: 10.1111/jsr.14276 (PMC11744252; doi:10.1111/jsr.14276)
Supplement: Supplementary file 1 — DATA S1. Supporting Information. [file JSR-34-e14276-s001.docx]

# SUPPLEMENTARY MATERIAL

|  | **Page** |
| --- | --- |
| Supplementary Methods |  |
| Data quality checks | [21](#_Supplementary_Methods) |
| Detailed procedures | [21](#_Detailed_procedures) |
|  |  |
| **Supplementary Figures** |  |
| Figure S1: Density plots depicting the distribution of observed and imputed data | [22](#Figure S1. Density plots depicting the distribution of variables of observed data (blue) and imputed data (red). The density plots provide a visual representation of the estimated probability density function for each variable, showing the shape and sprea) |
| Figure S2: Correlation matrix of all dynamics measures | [23](#_Figure_S2._Correlation) |
| Figure S3: Frequency plots insomnia severity index scores | [24](#Figure S3. Frequency plot of Insomnia Severity Index (ISI) scores. Dotted lines indicate the scale’s cut-off score, providing a reference for clinically significant insomnia levels. 0-7 = no clinically significant insomnia, 8-14 = subthreshold insomnia le) |
| Figure S4: GAMM model comparisons | [25](#Figure S4. GAMM model comparisons for each symptom. Lower AIC scores indicate better model fit while considering model complexity. The green columns indicate the better model based on Chi-square test of the fREML score (fast restricted maximum likelihood)) |
|  |  |
| **Supplementary Tables** |  |
| Table S1: GAMM results for predicting changes in anxiety or nervousness across the day from insomnia severity index (ISI). | [26](#_Table_S1._GAMM) |
| Table S2: GAMM results for predicting changes in stress across the day from insomnia severity index (ISI). | [26](#_Table_S2._GAMM) |
| Table S3: GAMM results for predicting changes in sleepiness across the day from insomnia severity index (ISI). | [26](#_Table_S3._GAMM) |
| Table S4: GAMM results for predicting changes in feeling down across the day from insomnia severity index (ISI). | [27](#_Table_S4._GAMM) |

# Supplementary Methods

## Data quality checks

For data quality purposes, two attention checks and one honesty check were included in the baseline session: “Please rate the response alternative 'agree (9)' for this question”; “Please answer 100”; and “Have you been completely honest in your answers?”. Data of participants who failed more than one quality check (*n* = 6) were excluded before analysis.

## Detailed procedures

Participants were divided into two groups for day 2 assessments during which they rated arousal symptoms six times across the day. Participants were instructed to complete the first rating on day 2 between 08:00-09:00, allowing for a self-chosen starting time to minimize the study’s impact on sleep duration. Subsequent ratings were separated by approximately three hours. Group 1 were sent notifications at 10:00, 13:00, 16:00, 19:00, and 22:00, and group 2 at 11:00, 14:00, 17:00, 20:00, and 23:00. The one-hour shift between the groups provided a larger number of different timepoints across the day. A subgroup of 65 participants completed five diurnal sessions separated by four hours instead of three, between 08:00-00:00. Participants were encouraged to start each assessment at the instructed time, but each respective session could be completed up until after that. Importantly, all data were time stamped and the time stamp was used for the analysis. Following the arousal symptom ratings, a brief cognitive test battery was completed (results not reported here). Each assessment took about 15 minutes to complete.

**Supplementary Figures**

## Figure S1. Density plots depicting the distribution of variables of observed data (blue) and imputed data (red). The density plots provide a visual representation of the estimated probability density function for each variable, showing the shape and spread of the imputed values. The value (i.e., ratings) range from 0 to 8.

## Figure S2. Correlation matrix showing Pearson correlation coefficients between all measures of dynamics (mean, MSSD, SD, and ACF); MSSD = mean squared successive differences; SD = standard deviation; ACF = autocorrelation function.

##
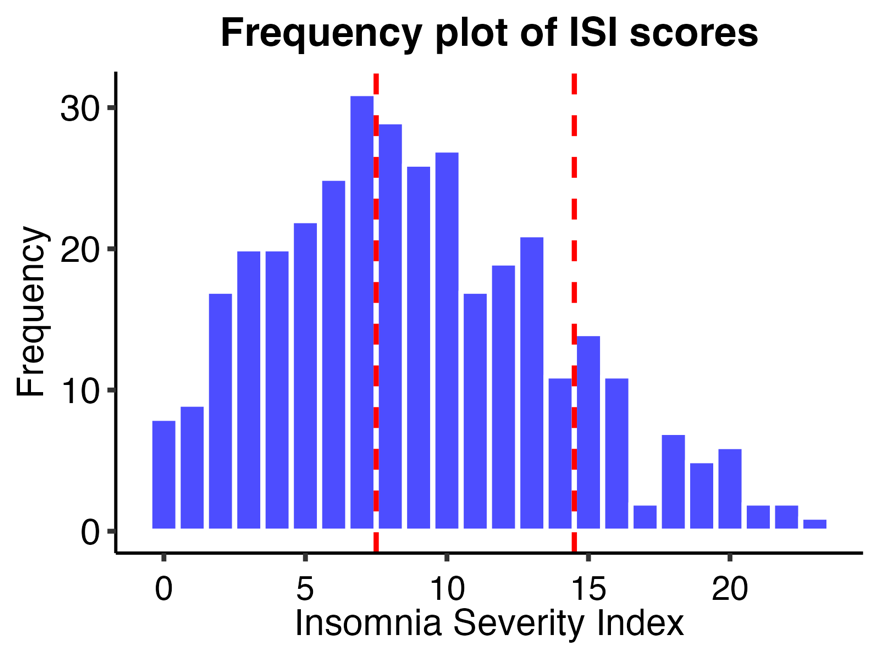
Figure S3. Frequency plot of Insomnia Severity Index (ISI) scores. Dotted lines indicate the scale’s cut-off score, providing a reference for clinically significant insomnia levels. 0-7 = no clinically significant insomnia, 8-14 = subthreshold insomnia levels, 15 or higher = moderate to severe clinical levels of insomnia.

## Figure S4. GAMM model comparisons for each symptom. Lower AIC scores indicate better model fit while considering model complexity. The green columns indicate the better model based on Chi-square test of the fREML score (fast restricted maximum likelihood). The main effects model contains a random intercept for participant ID and main effects of time-of-day and ISI score. The interaction effect model adds an interaction between time-of-day and ISI score.

**Supplementary Tables**

## Table S1. GAMM results for predicting changes in anxiety or nervousness across the day from insomnia severity index (ISI).

| *Anxious or nervous* | | | | |
| --- | --- | --- | --- | --- |
| Parametric coefficients | Estimate | SE | T value | p-value |
| Intercept | 0.38 | 0.07 | 5.41 | 6.18e-08*** |
|  |  |  |  |  |
| Smooth terms | EDF | RefDF | F-value | p-value |
| ID | 300.14 | 363 | 2127.66 | <2e-16*** |
| Time-of-day | 0.90 | 9 | 13.10 | 0.000*** |
| ISI | 1.10 | 19 | 3800.34 | <2e-16*** |
| Time-of-day x ISI | 7.51 | 22 | 62.31 | 4.35e-06*** |

Note. EDF (Effective Degrees of Freedom) indicates the flexibility of the smooth term and RefDF (Reference Degrees of Freedom) represents the DF used by the smoother.

## Table S2. GAMM results for predicting changes in stress across the day from ISI.

| *Stressed* | | | | |
| --- | --- | --- | --- | --- |
| Parametric coefficients | Estimate | SE | T value | p-value |
| Intercept | 0.65 | 0.06 | 10.74 | <2e-16*** |
|  |  |  |  |  |
| Smooth terms | EDF | RefDF | F-value | p-value |
| ID | 305.06 | 363 | 2134.59 | <2e-16*** |
| Time-of-day | 4.29 | 9 | 41.35 | 1.92e-95*** |
| ISI | 0.98 | 19 | 16773.82 | <2e-16*** |

Note. EDF (Effective Degrees of Freedom) indicates the flexibility of the smooth term and RefDF (Reference Degrees of Freedom) represents the DF used by the smoother.

## Table S3. GAMM results for predicting changes in sleepiness across the day from ISI.

| *Sleepy* | | | | |
| --- | --- | --- | --- | --- |
| Parametric coefficients | Estimate | SE | T value | p-value |
| Intercept | 4.19 | 0.09 | 46.83 | <2e-16*** |
|  |  |  |  |  |
| Smooth terms | EDF | RefDF | F-value | p-value |
| ID | 251.94 | 363 | 2.29 | <2e-16*** |
| Time-of-day | 4.43 | 9 | 6.97 | <2e-16*** |
| ISI | 0.81 | 19 | 0.33 | 0.001** |
| Time-of-day x ISI | 3.93 | 22 | 2.14 | <2e-16*** |

Note. EDF (Effective Degrees of Freedom) indicates the flexibility of the smooth term and RefDF (Reference Degrees of Freedom) represents the DF used by the smoother.

## Table S4. GAMM results for predicting changes in feeling down across the day from ISI.

| *Down* | | | | |
| --- | --- | --- | --- | --- |
| Parametric coefficients | Estimate | SE | T value | p-value |
| Intercept | 0.51 | 0.06 | 8.36 | <2e-16*** |
|  |  |  |  |  |
| Smooth terms | EDF | RefDF | F-value | p-value |
| ID | 298.81 | 363 | 1928.26 | <2e-16*** |
| Time-of-day | 3.32 | 9 | 47.69 | <2e-16*** |
| ISI | 0.99 | 19 | 15016.99 | <2e-16*** |

Note. EDF (Effective Degrees of Freedom) indicates the flexibility of the smooth term and RefDF (Reference Degrees of Freedom) represents the DF used by the smoother.
